# Comparing Efficacy and Safety of Bridging Therapy Versus Endovascular Thrombectomy in Acute Basilar Artery Occlusion: A Systematic Review and Meta‐Analysis

**DOI:** 10.1002/brb3.71277

**Published:** 2026-02-24

**Authors:** Muhammad Hassan Waseem, Zain ul Abideen, Aiman Waheed, Sanan Rasheed, Muneeba Ahsan, Rimsha Adnan, Muhammad Wajih Ansari, Rowaid Ahmad, Zara Fahim, Pawan Kumar Thada, Brandon Lucke‐Wold, Adam A. Dmytriw

**Affiliations:** ^1^ Allama Iqbal Medical College Lahore Pakistan; ^2^ King Edward Medical University Lahore Pakistan; ^3^ Rawalpindi Medical University Rawalpindi Pakistan; ^4^ Dow University of Health Sciences Karachi Pakistan; ^5^ University of Texas Medical Branch Galveston Texas USA; ^6^ Sotang Primary Hospital Solukhumbu Nepal; ^7^ University of Florida Florida USA; ^8^ Neuroendovascular Program, Massachusetts General Hospital & Brigham and Women's Hospital Harvard Medical School Boston Massachusetts USA; ^9^ Neurointerventional & Neuroanalytics Collaboration (NAN‐C), School of Medicine Toronto Metropolitan University Toronto Ontario Canada; ^10^ Nuffield Department of Surgical Sciences, Medical Sciences Division University of Oxford Oxford UK

**Keywords:** acute ischemic stroke, basilar artery occlusion, bridging therapy, endovascular thrombectomy, intravenous thrombolysis, meta‐analysis

## Abstract

**Background:**

Basilar artery occlusion (BAO), a rare and severe stroke, causes high morbidity and mortality. This meta‐analysis aims to compare bridging therapy, including endovascular thrombectomy (EVT) with intravenous thrombolysis (IVT), versus EVT alone in BAO.

**Methods:**

PubMed, Cochrane Central, and ScienceDirect were searched until May 2025. The risk ratios (RRs) and 95% confidence intervals (CIs) were combined using a random effects model in Review Manager software. The quality assessment was conducted using the Cochrane Risk of Bias (RoB 2.0) and the Newcastle–Ottawa scale. Publication bias was assessed visually via funnel plots and statistically using Egger's regression test. This review's protocol was registered on PROSPERO with the ID: CRD420251108752.

**Results:**

A total of 14 studies, including 11 observational studies and 3 randomized controlled trials, with 3745 participants, were analyzed. Bridging therapy was associated with a significantly higher likelihood of achieving functional independence (modified Rankin Scale (mRS) ≤ 2) (RR = 1.27; 95%CI: [1.13, 1.43]; *p *< 0.0001; *I* ^2^ = 23%) and independent ambulation (mRS ≤ 3) (RR = 1.10; 95%CI: [1.01, 1.20]; *p* = 0.02; *I* ^2^ = 0%). The mortality risk was also significantly lower in bridging therapy (RR = 0.83; 95%CI: [0.75, 0.93]; *p* = 0.001; *I* ^2^ = 0%). The successful recanalization endpoint was found to be comparable between the bridging therapy and EVT alone arms (RR = 1.00; 95%CI: [0.97, 1.03]; *p* = 0.99; *I* ^2^ = 0%). Similarly, there was no significant difference between the intervention and control groups in spontaneous intracranial hemorrhage (sICH) (RR = 0.99; 95%CI: [0.71, 1.39]; *p* = 0.97; *I* ^2^ = 0%).

**Conclusion:**

Our meta‐analysis supports administering IVT before EVT for BAO, showing benefits in functional outcomes and mortality without increasing hemorrhage risk, although successful recanalization was similar in both groups.

## Graphical abstract

The meta‐analysis compares bridging therapy to endovascular thrombectomy in acute basilar artery occlusion, finding that EVT+IVT provides significant functional benefits, lowers mortality risk, and does not increase hemorrhage risk compared to EVT alone, with similar recanalization success rates for both treatments.

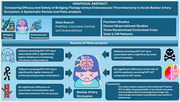

## 1 Introduction

Basilar artery occlusion (BAO) is a rare but life‐threatening form of ischemic stroke, representing nearly 1% of all strokes and about 27% of posterior circulation strokes. It occurs more frequently in males, with a 2:1 ratio (Reinemeyer et al. 2023; Schonewille et al. 2009). It is associated with high mortality and poor functional outcomes, with up to 80% of patients left severely disabled or deceased if not treated (Caplan's Stroke: A Clinical Approach 2016). Endovascular treatment (EVT) has become the mainstay for large vessel occlusions (LVO), especially in the anterior circulation, but its role in posterior circulation strokes, including BAO, has been supported by studies and randomized trials (Waseem et al. 2025; Waseem Ul Abideen, Farhan, et al. 2025; Langezaal et al. 2021).

In anterior circulation stroke management, bridging therapy, defined as intravenous thrombolysis (IVT) followed by EVT, has been considered effective in improving early reperfusion and clinical outcomes (Mistry et al. 2017). The proposed benefit of bridging therapy in BAO lies in IVT's potential to lyse the clot before EVT or assist in disintegrating distal emboli not accessible by mechanical means (Lee et al. 2020). However, the clinical benefit of bridging over EVT alone remains uncertain, particularly in BAO, where evidence is mostly derived from observational studies and registry data (Yao et al. 2022).

While some studies have reported improved rates of successful recanalization and functional independence with bridging therapy, others have failed to demonstrate significant differences in outcomes. Additionally, using IVT could raise the risk of symptomatic intracranial hemorrhage (sICH) and may postpone the start of EVT (Zi et al. 2020). The decision to administer IVT before EVT is also influenced by time windows, contraindications, and institutional protocols, contributing to wide variations in clinical practice.

Given the potential impact on morbidity and mortality in BAO, it is imperative to evaluate whether bridging therapy confers additional benefit over EVT alone. To date, the literature lacks consensus, and a comprehensive meta‐analysis is needed to guide clinical decision‐making. This meta‐analysis compares EVT with IVT versus EVT alone in patients with BAO.

## 2 Methods

This systematic review and meta‐analysis adhered to the Preferred Reporting Items for Systematic Reviews and Meta‐Analyses (PRISMA) guidelines (Page et al. 2021) and was conducted in accordance with the Handbook for Systematic Reviews of Interventions by Cochrane (Higgins et al. 2019). The protocol for this review was registered on PROSPERO under the ID: CRD420251108752.

### 2.1 Search Strategy

A comprehensive literature search was conducted in PubMed, Cochrane Central, and ScienceDirect to identify relevant studies published through May 2025. The MeSH terms used were “stroke,” “thrombosis,” “basilar artery,” “thrombolytic therapy,” and “thrombectomy.” The keywords used included “ischemic stroke,” “cerebrovascular occlusion,” “cerebrovascular accident,” “cerebral stroke,” “thrombolysis,” “fibrinolytic therapy,” and “endovascular therapy.” These MeSH terms and keywords were combined with the Boolean operators “OR” and “AND.” The bibliographies of included studies and reviews were also screened to capture additional records. The details of individual search strategies used in different electronic databases are given in Table S1.

### 2.2 Study Selection and Eligibility Criteria

**FIGURE 1:**
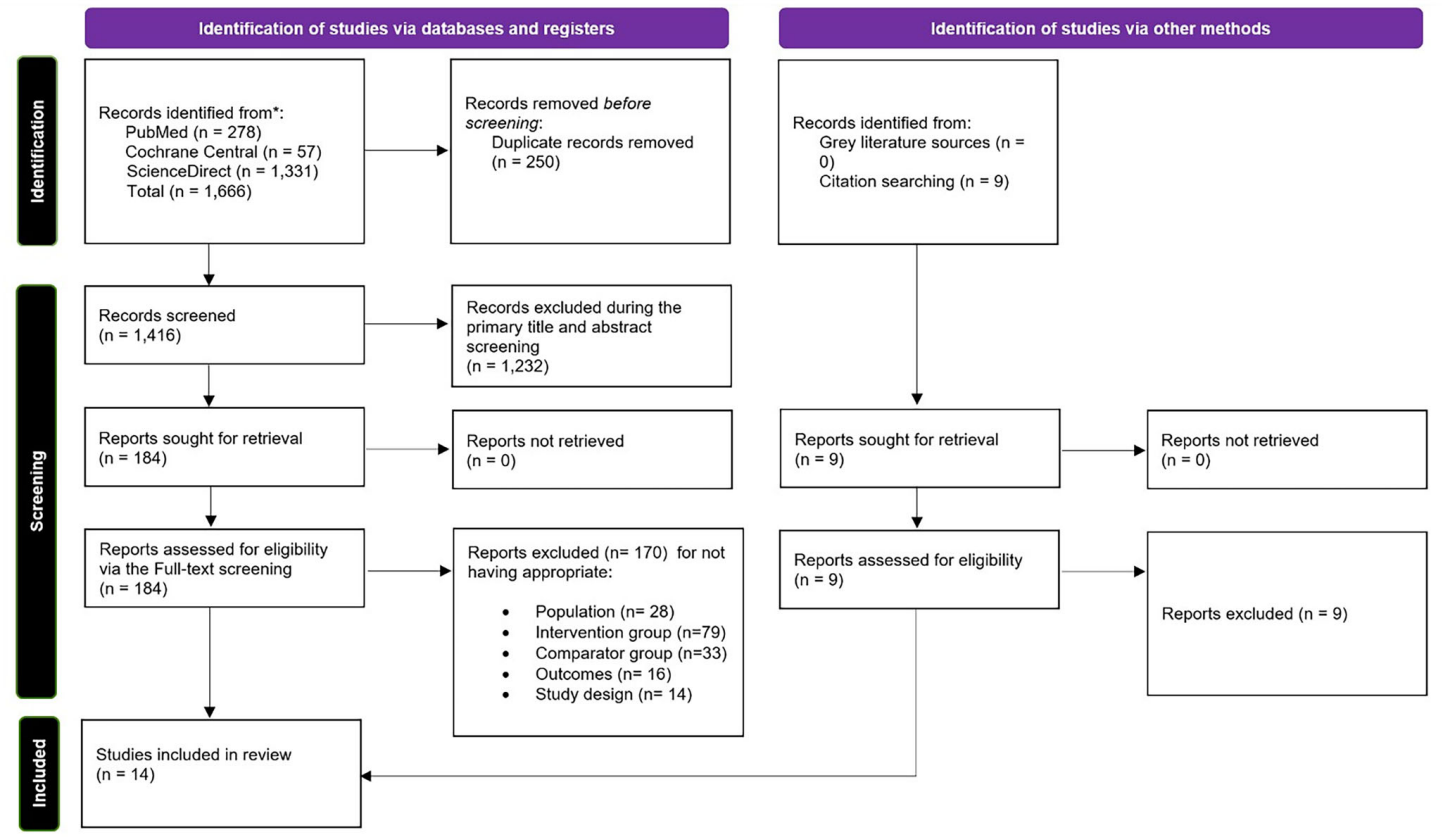
PRISMA flowchart of the study selection process.

Two authors (A.W. and S.R.) independently conducted the initial search, and the retrieved articles were imported into EndNote. After removing duplicates, the remaining articles were screened by titles and abstracts. The articles that remained after the primary screening were subjected to a secondary screening via full‐text review. Any disagreements were resolved by discussion with a third reviewer (M.H.W.). The detailed process for selecting studies is shown in the PRISMA flowchart (Figure 1).

Eligible studies are randomized controlled trials (RCTs) or observational studies that enrolled adult patients (aged ≥18 years) with radiologically confirmed acute BAO and compared outcomes between EVT+IVT and EVT alone. Included studies reported at least one of the outcomes being analyzed. Non‐comparative studies, pediatric populations, and studies without treatment‐group‐specific outcomes were excluded. Additionally, due to the lack of consensus on the timing of EVT initiation in BAO, studies without a specified time limit were included.

### 2.3 Data Extraction and Outcomes Definition

Two authors (Z.U.A. and M.A.) independently extracted data into an Excel sheet. The baseline characteristics extracted were study ID, study design, location, sample size, age, percentage of males, median National Institutes of Health Stroke Scale (NIHSS) score, occlusion sites, and history of previous stroke.

The endpoints extracted were functional independence, independent ambulation, successful recanalization, mortality, and sICH. Functional independence was defined as a modified Rankin score (mRS) of ≤ 2, whereas independent ambulation was defined as an mRS score of ≤ 3. The successful recanalization was defined as a modified treatment in cerebral ischemia (TICI) score of 2b‐3.

### 2.4 Quality Assessment

The Cochrane Risk of Bias (RoB) 2.0 tool was used to evaluate RCTs (Sterne et al. 2019), whereas the observational studies were evaluated with the Newcastle–Ottawa scale (Gláucia et al. 2013). RoB 2.0 has five domains: bias from randomization, deviations from interventions, missing data, outcome measurement, and result selection. NOS judges studies based on selection, comparability, and outcome.

### 2.5 Statistical Analysis

Statistical analyses were conducted with Review Manager (RevMan version 5.4.1). Risk ratios (RRs) with 95% confidence intervals (CIs) were calculated for dichotomous outcomes. A random‐effects model was employed to address heterogeneity, evaluated using the Cochrane *Q* test and Higgins *I* ^2^ statistics (Higgins et al. 2003). A *p* value of less than 0.05 was considered statistically significant. No significant heterogeneity was detected across the analyzed outcomes; thus, further heterogeneity analyses were not needed. Publication bias was assessed visually using funnel plots and statistically using Egger's test in comprehensive meta analysis (CMA) version 3.0. GRADE assessed the certainty of evidence for all outcomes using GRADEpro GDT (Guyatt et al. 2011).

## 3 Results

The initial database search yielded 1666 records. After removing 250 duplicates, 1416 unique records remained for preliminary screening. Title and abstract screening led to the exclusion of 1232 records. The remaining 184 articles were then thoroughly assessed against predefined inclusion criteria. Of these, 170 were excluded, and ultimately, 14 studies (Langezaal et al. 2021; Guo et al. 2024; Knapen et al. 2024; Feil et al. 2023; Kaneko et al. 2019; Singer et al. 2015; Siow et al. 2022; Nappini et al. 2021; Nie et al. 2022; Yang et al. 2025; Han et al. 2025; Jovin et al. 2022; Maïer et al. 2023; Uno et al. 2017) met the inclusion criteria and were incorporated into the meta‐analysis. The PRISMA flow diagram (Figure 1) illustrates the full study selection process.

### 3.1 Included Studies Characteristics

This systematic review and meta‐analysis incorporated 14 studies (11 observational studies and 3 RCTs), involving 3745 participants: 1422 receiving EVT combined with IVT and 2323 receiving EVT alone. Patient ages ranged from 60.5 to 72 years, and the median NIHSS score ranged from 13 to 28. Table 1 presents the baseline characteristics of the included studies.

**TABLE 1:** Baseline characteristics of the included studies.

			Sample size		Age		Male *n* (%)		Pre NIHSS		Occlusion site *n* (%)				Previous stroke *n* (%)	
Study ID	Study design	Location	EVT	EVT+IVT	EVT	EVT+IVT	EVT	EVT+IVT	EVT	EVT+IVT	Distal	Middle	Proximal	VA‐V4	EVT	EVT+IVT
Knapen et al. 2024	OS	Netherlands	123	125	69 (56–76)	62 (52–71)	64 (52)	78 (62)	17 (9.3–29)	15 (7.5–31)	EVT = 38/120 (32) EVT + IVT = 54/123 (44)	—	—	EVT = 6/120 (5.0) EVT + IVT = 8/123 (6.5)	27/120 (23)	19/125 (15)
Guo et al. 2024	OS	China	528	119	65.0 (56.0–73.0)	62.0 (56.0–71.0)	394 (74.6)	89 (74.8)	26.0 (16.0–33.0)	28.0 (18.0–34.0)	EVT = 177 (33.5) EVT + IVT = 45 (37.8)	EVT = 160 (30.3) EVT + IVT = 35 (29.4)	EVT = 87 (16.5) EVT + IVT = 20 (16.8)	EVT = 104 (19.7) EVT + IVT = 19 (16)	410 (77.7)	97 (81.5)
Feil et al. 2023	OS	Germany	355	285	72.2 ± 13.3		363(56.7)		17 (8, 27)		—	—	—	—	—	—
Kaneko et al. 2019	OS	Japan	27	21	—	—	35		—	—	18(37.5)	17(35.4)	12(25)	—	—	—
Singer et al. 2015	OS	Germany	61	87	71 (61–77)		96 (65)		20 (9–28)		36 (26)	57 (41)	46 (33)	—	—	—
Uno et al. 2017	OS	Japan	16	18	72 (66–77)		23 (68)		23 (68)		19 (56)	11 (32)	4 (12)	—	11 (32)	
Siow et al. 2022	OS	Europe, Asia	195	127	66.3±14.0	69.4±14.0	133 (68.2)	73 (57.5)	17 (8–26)	14 (8–22)	—	—	—	—	18/129 (14.0)	16/86 (18.6)
Nappini et al. 2021	OS	Italy	298	166	67.9 (±13.22)	67.1 (±13.42)	196 (66)	107 (65)	18 (10–31)	17 (9–25)	187 (40)	91 (20)	72 (16)	—	—	—
Nie et al. 2022	OS	China	241	69	61.63±11.40	60.54±9.11	186 (77.18)	54 (78.26)	21 (12–27)	20 (9–27)	EVT = 30 (12.45) EVT + IVT = 12 (17.39)	EVT = 78 (32.37) EVT + IVT = 21 (30.43)	EVT = 44 (18.26) EVT + IVT = 13 (18.84)	EVT = 89 (36.93) EVT + IVT = 23 (33.33)	67 (27.80)	13 (18.84)
Yang et al. 2024	OS	China	65	88	67.7±13.0	67.3±12.0	37 (56.9)	58 (65.9)	22 (10,35)	26 (15,35)	—	—	—	—	12 (18.5)	15 (17.0)
Han et al. 2025	RCT	China	154	67	65.8±11.4	66.0±10.5	108(70)	40(60)	24.5 (15.0,35.0)	24.0 (16.0,35.0)	EVT = 50(32) EVT + IVT = 22(33)	EVT = 37(24) EVT + IVT = 23(35)	EVT = 50(32) EVT + IVT = 18(27)	EVT = 17(11) EVT + IVT = 3 (5)	43(28)	11(16)
Jovin et al. 2022	RCT	China	95	15	64.2±9.6		80 (73)		20 (15–29)		13/107 (12)	40/107 (37)	53/107 (50)		—	—
Langezaal et al. 2021	RCT	Netherlands, Germany, Brazil	33	121	66.8±13.1		100(64.9)		21		—	—	—	—	11/154 (7.1)	
Maier et al. 2023	OS	France	132	114	66.8 ±15	66.7 ±16	84(63.6)	77(67.5)	13(11)	14(14)	—	—	—	—	—	—

*Note*: Data is reported as mean (standard deviation) and median (range).

Abbreviations: EVT, endovascular thrombectomy; IVT, intravenous thrombolysis; NIHSS, National Institute of Health Stroke Scale; OS, observational studies; RCT, randomized controlled trials.

### 3.2 Quality Assessment

**FIGURE 2:**
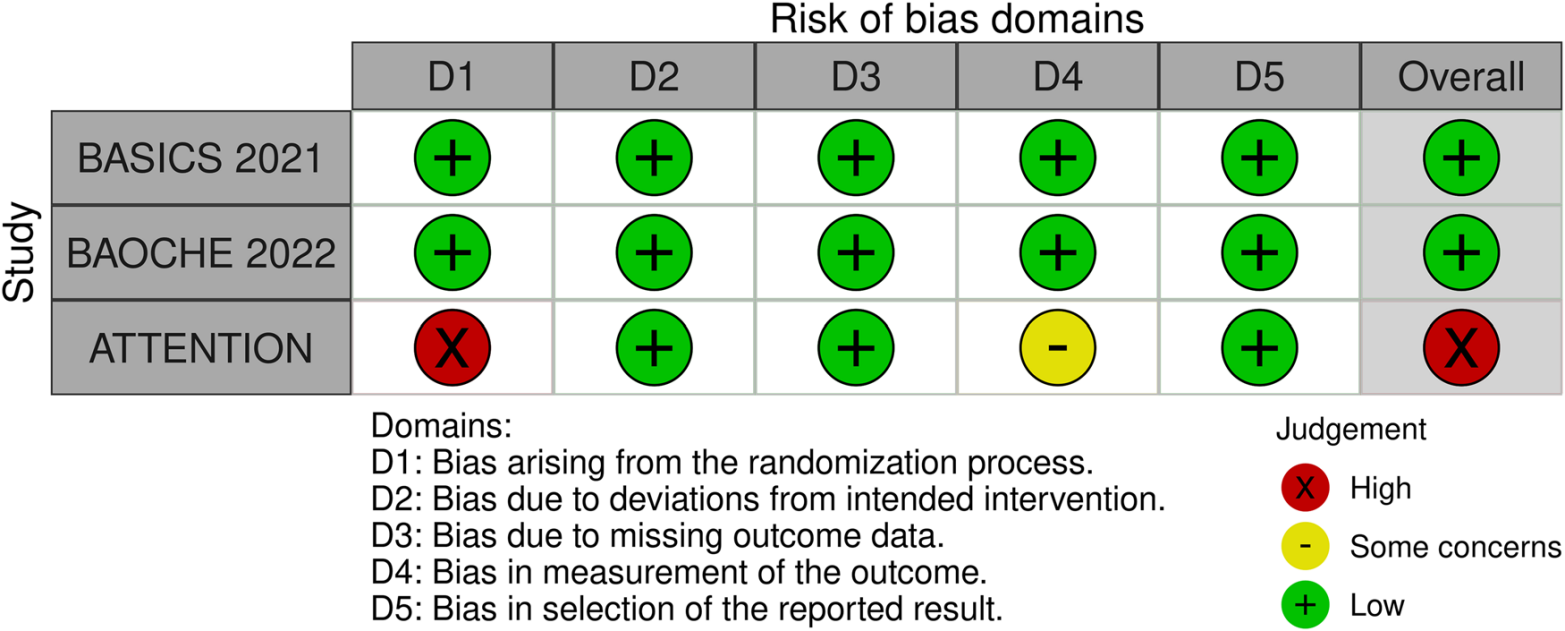
Risk of Bias traffic plot.

The two RCTs (BASICS 2021 (Langezaal et al. 2021) and BAOCHE 2022 (Jovin et al. 2022)), assessed using the RoB 2.0 tool, showed low risk of bias across all domains except the ATTENTION trial (Han et al. 2025), which showed a high risk of bias due to bias arising from the randomization process and measurement of outcomes. Observational studies were evaluated using NOS, with scores ranging from 6 to 9. Guo et al. (2024) and Nie et al. (2022) achieved full scores, indicating high quality. Most other studies demonstrated good selection and comparability, though a few had limitations in outcome assessment and follow‐up. Overall, the studies were of moderate to high quality. The RoB 2.0 traffic plot and the NOS quality assessment are shown in Figure 2 and Table S2.

### 3.3 Outcomes

The summary of the meta‐analysis is presented in Table 2.

**TABLE 2:** Summary of the meta‐analysis.

		Sample size			95% confidence interval			Heterogeneity					
Endpoint	Number of studies	EVT	EVT+IVT	Effect size (RR)	Lower limit	Upper limit	*p* value	Tau^2^	Chi^2^	*df*	*p*	*I* ^2^ (%)	Egger's regression intercept *p* value
Functional independence	13	2258	1264	1.27	1.13	1.43	<0.0001	0.01	15.67	12	0.21	23	0.5625
Independent ambulation	10	1931	1169	1.10	1.01	1.20	0.02	0	4.64	9	0.86	0	0.0018
Successful recanalization	10	2145	1133	1.00	0.97	1.03	0.99	0	6.23	9	0.72	0	0.6152
Mortality	10	2217	1138	0.83	0.75	0.93	0.001	0	5.58	9	0.78	0	0.6818
Spontaneous intracerebral hemorrhage	9	1781	848	0.99	0.71	1.39	0.97	0	3.47	8	0.90	0	0.4797

Abbreviations: df, degree of freedom; EVT, endovascular thrombectomy; IVT, intravenous thrombolysis; RR, risk ratio.

### 3.4 Functional Independence

**FIGURE 3:**
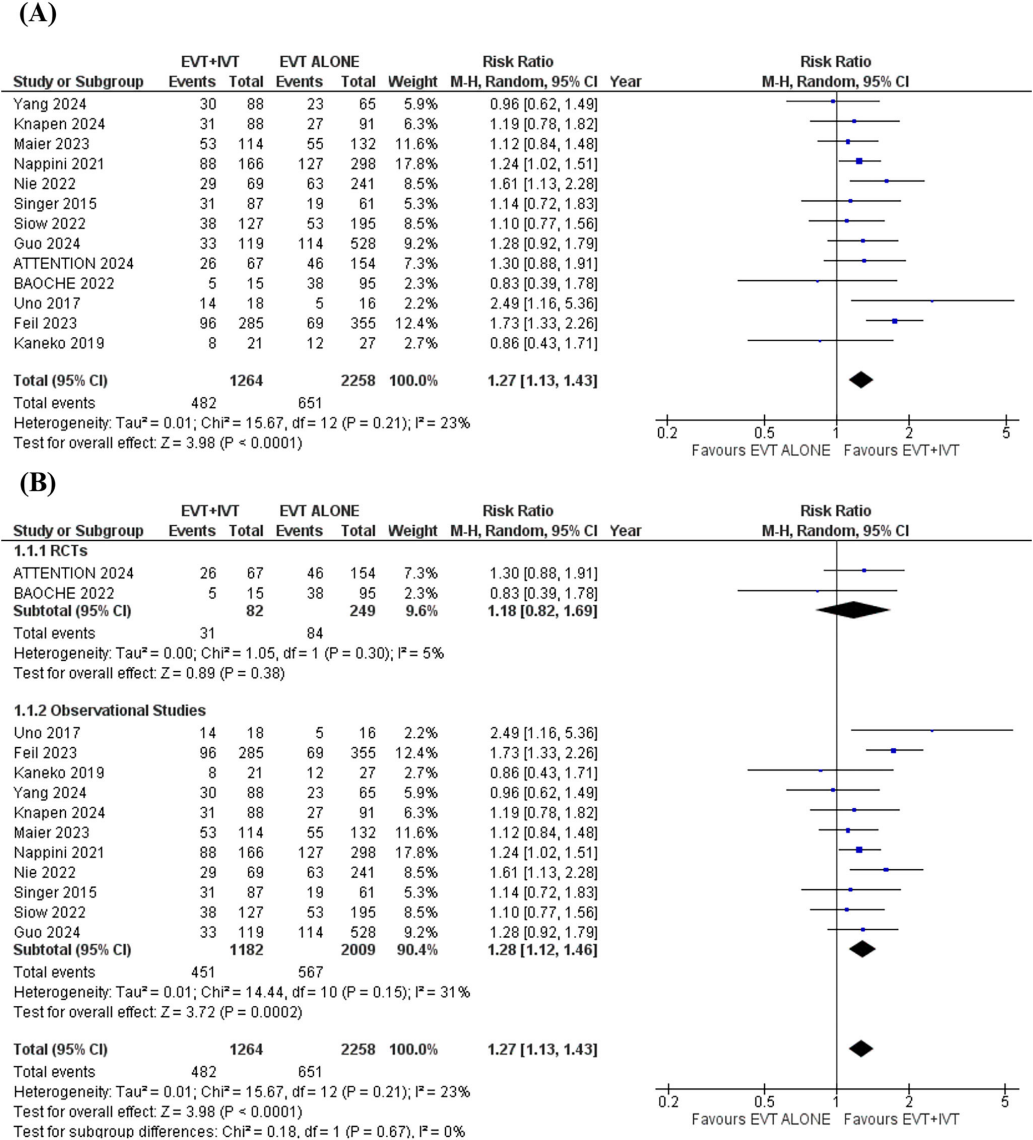
Functional independence forest plot.

Thirteen studies reported data regarding functional independence. Patients receiving EVT+IVT were associated with a significantly higher likelihood of achieving functional independence compared to those receiving EVT alone (RR = 1.27; 95%CI:[1.13 to 1.43]; *p* < 0.0001; *I* ^2^ = 23%) (Figure 3A).

### 3.5 Independent Ambulation

**FIGURE 4:**
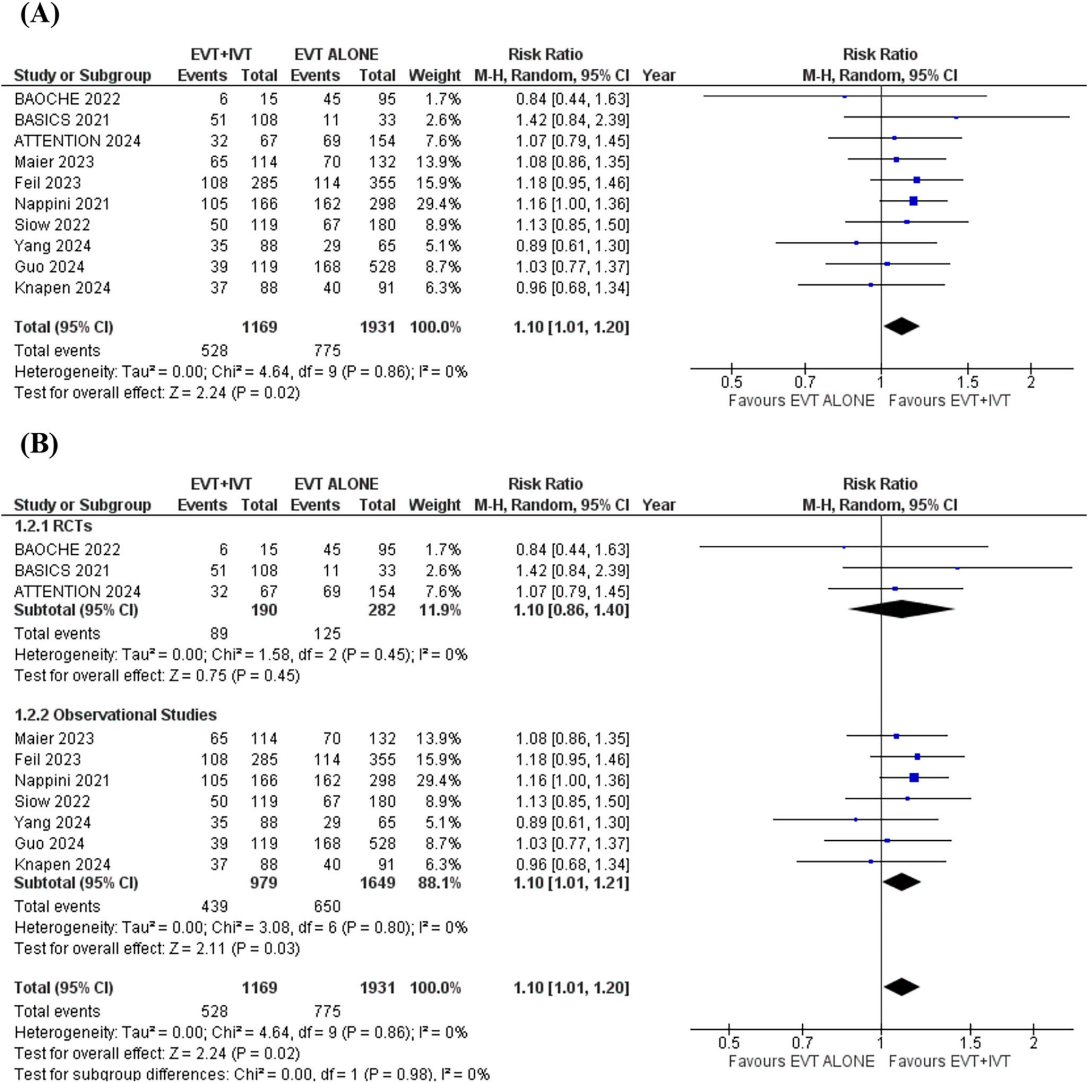
Independent ambulation forest plot.

Ten studies reported this outcome. In the pooled analysis, patients receiving EVT+IVT had a significantly higher chance of achieving independent ambulation compared to those receiving EVT alone (RR = 1.10; 95% CI: [1.01 to 1.20]; *p* = 0.02; *I* ^2^ = 0%) (Figure 4A).

### 3.6 Successful Recanalization

**FIGURE 5:**
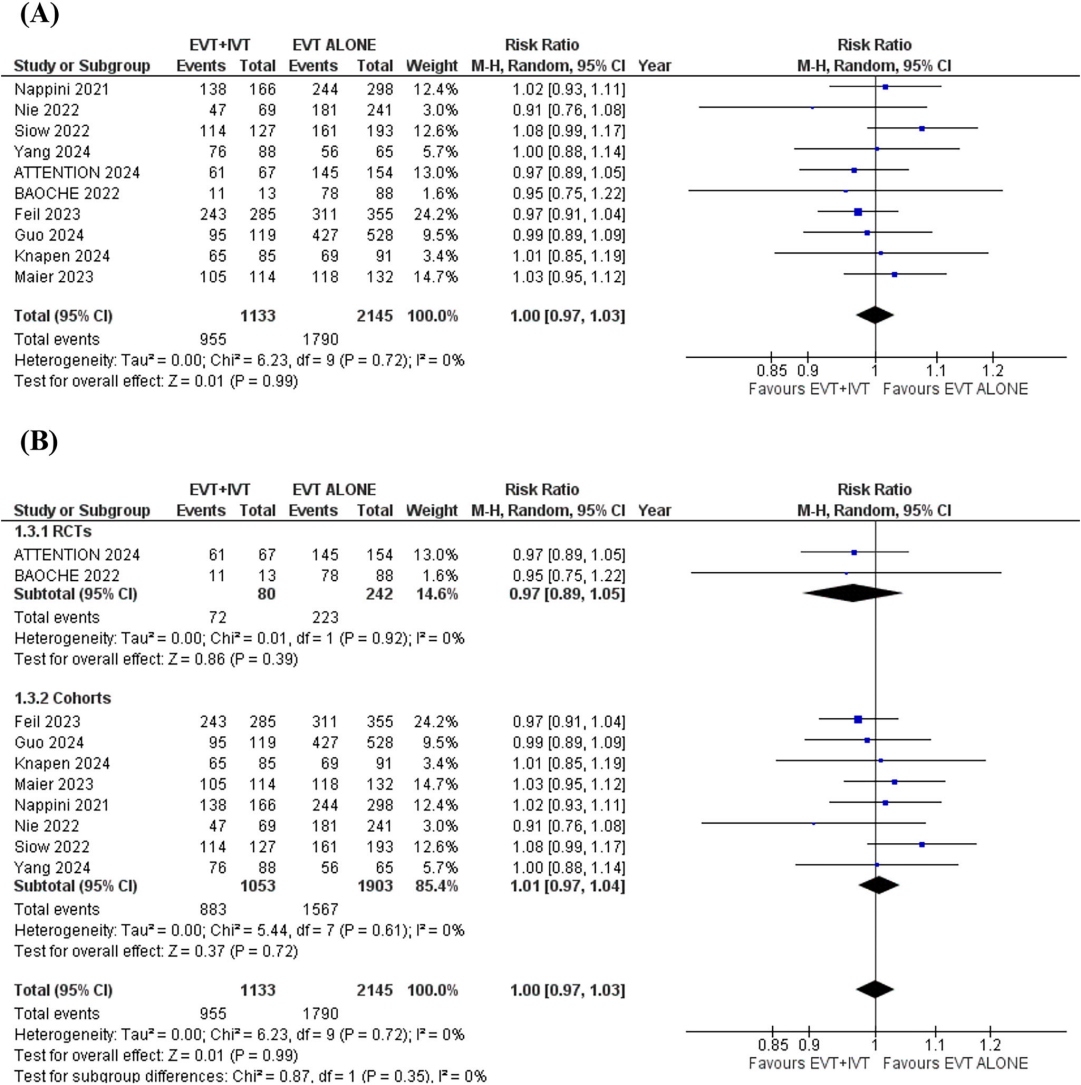
Successful recanalization forest plot.

Ten studies reported the outcome of successful recanalization. In the pooled analysis, no significant difference in successful recanalization was observed between the two groups (RR = 1.00; 95% CI: [0.97 to 1.03]; *p* = 0.99; *I* ^2^ = 0%) (Figure 5A).

### 3.7 Mortality

**FIGURE 6:**
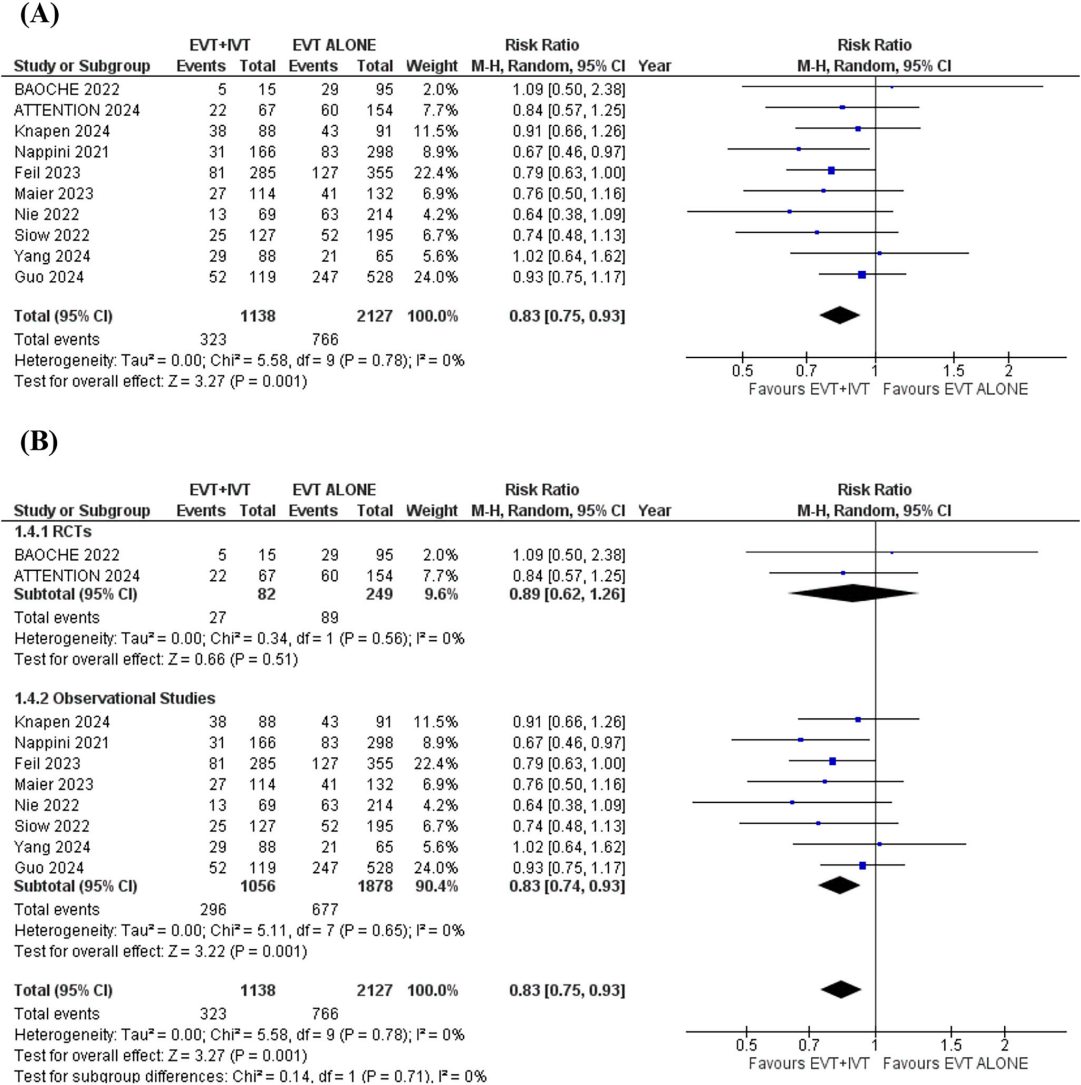
Mortality forest plot.

Ten studies were analyzed for this outcome. In the pooled analysis, patients receiving EVT+IVT were associated with a significantly lower risk of mortality compared to those receiving EVT alone (RR = 0.83; 95%CI: [0.75 to 0.93]; *p* = 0.001; *I* ^2^ = 0%) (Figure 6A).

### 3.8 Symptomatic Intracranial Hemorrhage

**FIGURE 7:**
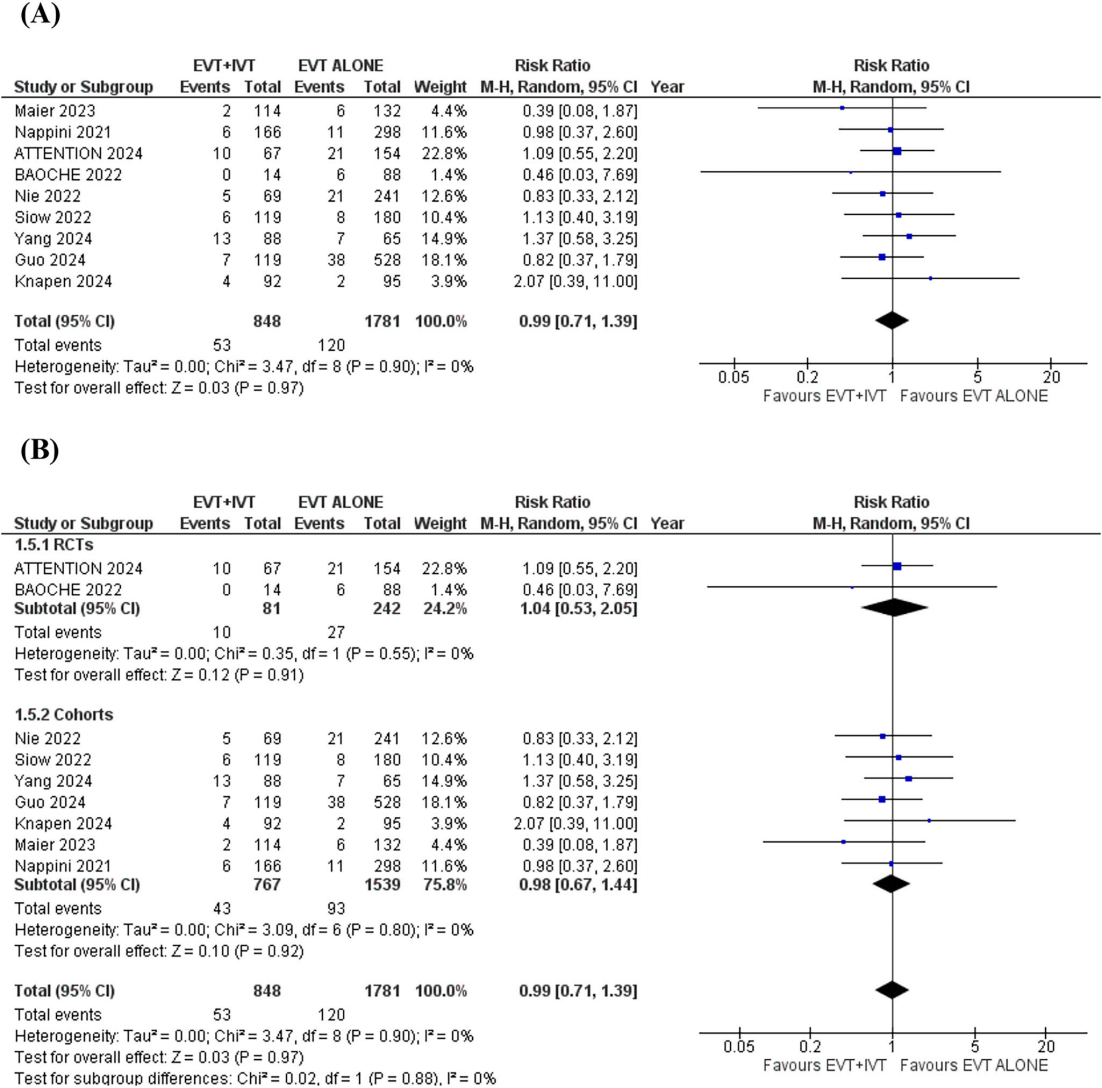
Spontaneous intracranial hemorrhage forest plot.

Nine studies reported the outcome of sICH. In the pooled analysis, there was no significant difference in the risk of sICH in patients receiving EVT+IVT compared to EVT alone (RR = 0.99; 95% CI:[0.71 to 1.39]; *p* = 0.97; *I* ^2^ = 0%) (Figure 7A).

### 3.9 Subgroup Analysis

Subgroup analysis was conducted based on study design (RCTs or observational cohorts). The results remained consistent across all outcomes after subgrouping (Figures 5B and 7B). However, in the RCTs subgroup, no significant differences were found for functional independence (RR: 1.18; 95% CI: 0.82 to 1.69; *p* = 0.38), independent ambulation (RR: 1.10; 95% CI: 0.86 to 1.40; *p* = 0.45), and all‐cause mortality (RR: 0.89; 95% CI: 0.62 to 1.26; *p* = 0.51). This lack of significance in the RCTs subgroup may be due to the smaller number and smaller sample sizes of the pooled RCTs, which reduced statistical power to detect significant differences (Figures 3, 4 and 6B). More large‐scale randomized trials are necessary to strengthen the evidence and confirm these results.

### 3.10 Publication Bias

Funnel plots were used to assess publication bias, revealing no asymmetry visually. This indicates no publication bias, a finding further supported by Egger's regression test (Figures S1–S6 and S8–S10), except for the outcome of independent ambulation, which showed significant publication bias on the Egger's regression analysis (Egger's regression intercept = −2.415; *p* = 0.0018) (Figure S7).

### 3.11 GRADE Assessment

The GRADE assessment evaluated the certainty of the evidence. The evidence from RCTs was of moderate certainty across all outcomes, whereas evidence from observational studies ranged from low to very low certainty. The GRADE summary of findings is provided in Table 3.

**TABLE 3:** GRADE summary of findings.

EVT+IVT compared to EVT alone for BAO					
Patient or population: BAO Intervention: EVT+IVT Comparison: EVT Alone					
	Anticipated absolute effects (95% CI)				
Outcomes	Risk with EVT Alone	Risk with EVT+IVT	Relative effect (95% CI)	№ of participants(studies)	Certainty of the evidence (GRADE)
Functional independence (mRS ≤ 2)—RCTs	337 per 1000	**398 per 1000** (277 to 570)	**RR 1.18** (0.82 to 1.69)	331 (2 RCTs)	⨁⨁⨁◯ Moderate^a^
Functional independence (mRS ≤ 2)—Observational Studies	282 per 1000	**361 per 1000** (316 to 412)	**RR 1.28** (1.12 to 1.46)	3191 (11 non‐randomized studies)	⨁⨁◯◯ Low
Independent ambulation (mRS ≤ 3)—RCTs	443 per 1000	**488 per 1000** (381 to 621)	**RR 1.10** (0.86 to 1.40)	472 (3 RCTs)	⨁⨁⨁◯ Moderate^a^
Independent ambulation (mRS ≤ 3)—Observational Studies	394 per 1000	**434 per 1000** (398 to 477)	**RR 1.10** (1.01 to 1.21)	2628 (7 non‐randomized studies)	⨁⨁◯◯ Low
Successful recanalization (modified treatment in cerebral ischemia (TICI) score 2b‐3)—RCTs	921 per 1000	**894 per 1000** (820 to 968)	**RR 0.97** (0.89 to 1.05)	322 (2 RCTs)	⨁⨁⨁◯ Moderate^a^
Successful recanalization (modified treatment in cerebral ischemia (TICI) score 2b‐3)—Observational Studies	823 per 1000	**832 per 1000** (799 to 856)	**RR 1.01** (0.97 to 1.04)	2956 (8 non‐randomized studies)	⨁◯◯◯ Very low^a^
Mortality—RCTs	357 per 1000	**318 per 1000** (222 to 450)	**RR 0.89** (0.62 to 1.26)	331 (2 RCTs)	⨁⨁⨁◯ Moderate^a^
Mortality—observational studies	360 per 1000	**299 per 1000** (267 to 335)	**RR 0.83** (0.74 to 0.93)	2934 (8 non‐randomized studies)	⨁⨁◯◯ Low
SICH (spontaneous intracerebral hemorrhage)—RCTs	112 per 1000	**116 per 1000** (59 to 229)	**RR 1.04** (0.53 to 2.05)	323 (2 RCTs)	⨁⨁⨁◯ Moderate^a^
SICH (spontaneous intracerebral hemorrhage)—observational studies	60 per 1000	**59 per 1000** (40 to 87)	**RR 0.98** (0.67 to 1.44)	2306 (7 non‐randomized studies)	⨁◯◯◯ Very low^a^

Abbreviations: CI, confidence interval; RR, risk ratio.

^a^The 95% confidence interval crosses 1.

## 4 Discussion

BAO remains one of the most severe forms of ischemic stroke, often resulting in devastating outcomes without effective intervention. While EVT has revolutionized management for LVO, the added benefit of IVT prior to EVT, known as “bridging therapy” for BAO, continues to generate debate. Our meta‐analysis of 14 studies evaluated the impact of EVT+IVT versus EVT alone on key outcomes, showing a higher likelihood of achieving functional independence and independent ambulation, and a lower mortality risk in the EVT+IVT group. In contrast, successful recanalization and sICH were comparable between the two groups. In the subgroup analysis based on the study design (cohorts and RCTs), no significant differences were observed in the RCTs subgroup for functional independence, independent ambulation, and all‐cause mortality. This lack of significance in the RCTs subgroup may be due to the smaller number and smaller sample sizes of the pooled RCTs, which reduced statistical power to detect significant differences. In the GRADE assessment, evidence from RCTs showed moderate certainty across all outcomes, whereas observational studies provided evidence of low certainty.

The significantly higher likelihood of achieving functional independence and independent ambulation, and a significantly lower risk of mortality in the EVT+IVT group, despite comparable successful recanalization rates between the two groups, may be attributed to factors beyond arterial reopening alone. While successful recanalization is necessary for arterial reopening and restoring blood flow, IVT may provide additional benefits by increasing microvascular perfusion and reducing the ischemic penumbra, thereby promoting better functional outcomes with lower mortality risk.

The finding of improved functional independence with EVT+IVT aligns with the results reported by Maïer et al. (2023), who analyzed a multicenter cohort and found a trend toward better functional outcomes in the EVT+IVT group, suggesting a potential benefit in certain populations, despite statistical insignificance in their cohort. Similarly, the BAOCHE trial by Tao et al. (2022) demonstrated a favorable shift in the mRS distribution in the EVT+IVT group, though statistical significance was not achieved for mRS ≤ 2 as a dichotomous outcome. In contrast, the BASICS trial by Langezaal et al. (2021) failed to demonstrate a functional advantage with IVT pretreatment, possibly due to high crossover rates and variable selection criteria.

The analysis of independent ambulation further supports the potential benefit of bridging therapy. Although the observed RR of 1.10 indicates a modest improvement, it was statistically significant. This aligns with data from Ren et al. (2018), who reported that pre‐treatment with IVT led to partial clot fragmentation and facilitated thrombectomy, potentially enhancing early ambulation and neurologic recovery. However, Maïer et al. (2023) noted that this effect might be confounded by earlier presentation times in the EVT+IVT group.

Regarding successful recanalization, no significant difference was observed between EVT+IVT and EVT alone in the pooled analysis. This finding is mirrored in a study by Mueller et al. (2017), who showed that prior IVT did not significantly affect angiographic success rates. The BAOCHE and BASICS trials similarly found no added benefit of IVT in terms of final TICI scores (Langezaal et al. 2021; Jovin et al. 2022). This lack of effect may be due to the nature of thrombi in the posterior circulation, which are often larger and more resistant to lytic therapy.

A notable and clinically meaningful finding in this meta‐analysis is the significantly lower mortality risk with EVT+IVT. This effect was particularly evident in observational studies. Nappini et al. (2021) reported lower mortality rates in the bridging group, suggesting that thrombolysis may contribute to microvascular reperfusion even when macrovascular recanalization is achieved through EVT. Ding et al. (2017) proposed that IVT could help dissolve distal emboli and reduce infarct volume, thus lowering the risk of fatal brainstem infarction—a plausible explanation for the survival benefit observed in our analysis.

However, not all studies agree. Nie et al. (2022), in their retrospective study of Chinese patients with BAO, observed no significant difference in mortality between EVT+IVT and EVT alone, raising questions about patient selection and stroke pathophysiology across different populations. The higher prevalence of intracranial atherosclerotic disease (ICAD) in Asian populations may reduce the effectiveness of IVT, as clots in these patients tend to be platelet‐rich and less responsive to alteplase (Krasteva et al. 2020). This geographic variability reinforces the need for individualized treatment strategies.

Safety concerns, particularly regarding sICH, were unfounded in this analysis. No significant difference was observed between EVT+IVT and EVT alone, aligning with the findings of large cohort studies such as those by Goyal et al. (2016) and Kaesmacher et al. (2019), which demonstrated similar bleeding risks between treatment strategies. A recent meta‐analysis by Jazayeri et al. (2024) also found no significant difference in the risk of sICH between the EVT+IVT and EVT alone arms in acute ischemic stroke, further supporting our findings. In the posterior circulation, where the brainstem's critical structures increase the risk of catastrophic hemorrhage outcomes, the absence of increased sICH provides reassurance to clinicians. A study by Desilles et al. (2015) provided valuable insight by showing that IVT may reduce thrombus adhesion and stiffness, thereby enhancing EVT success without significantly increasing hemorrhagic complications. Moreover, while clot migration is a theoretical concern with IVT, Ren et al. (2018) showed that such events rarely led to worse outcomes and were, in some cases, associated with improved procedural efficiency.

### 4.1 Strengths and Limitations

This meta‐analysis has several noteworthy strengths. The inclusion of both RCTs and observational studies provides a comprehensive evaluation of the efficacy and safety of EVT with or without IVT in acute BAO. A large sample size of 3745 patients adds statistical power and robustness to the findings. The subgroup analysis further improves interpretability by distinguishing outcomes based on study design.

However, this study is not without limitations. The predominance of observational studies introduces potential bias due to confounding variables and non‐randomized treatment allocation. Significant heterogeneity across studies in patient selection, stroke etiology (e.g., embolic vs. atherosclerotic), timing of interventions, and imaging modalities may limit the comparability of results. Geographic differences, particularly the higher prevalence of intracranial atherosclerosis in Asian cohorts, may also influence treatment response and reduce generalizability. Furthermore, not all studies reported detailed baseline perfusion imaging, limiting our ability to control for important prognostic factors. Another limitation of this meta‐analysis is the small number and sample size of included RCTs, which may have reduced statistical power to detect significant differences for some outcomes in the subgroup analysis by study design. Therefore, more large‐scale RCTs are needed to identify any significant differences and validate these findings. Despite these limitations, the study provides valuable evidence supporting the selective use of IVT before EVT in appropriately chosen patients with acute BAO.

## 5 Conclusion

Our meta‐analysis supports the use of bridging IVT in appropriately selected patients undergoing EVT for BAO. Significant benefits were observed in functional outcomes and mortality without an added risk of hemorrhage. These findings align with a growing body of data suggesting that IVT can complement EVT when administered judiciously. However, the comparable findings from RCTs remind us that patient selection and stroke etiology remain crucial variables. Future large‐scale RCTs are needed to refine these strategies further.

## Author Contributions

Study concept and design: Muhammad Hassan Waseem and Zain ul Abideen. Acquisition of data: Zain ul Abideen, Aiman Waheed, and Sanan Rasheed. Analysis and interpretation of data: Zain ul Abideen, Muneeba Ahsan, and Rowaid Ahmad. Drafting of the manuscript: Rowaid Ahmad, Zara Fahim, Muhammad Wajih Ansari, and Pawan Kumar Thada. Critical revision of the manuscript: Adam A. Dmytriw and Brandon Lucke‐Wold.

## Funding

The authors have nothing to report.

## Conflicts of Interest

The authors declare that they have no conflicts of interest.

## Supporting information




**Supplementary Material**: brb371277‐sup‐0001‐SuppMat.docx

## Data Availability

Data can be obtained upon reasonable request to the authors.
